# Early bacteremia following allogeneic hematopoietic stem cell transplantation without antibiotic prophylaxis: epidemiology and antimicrobial resistance

**DOI:** 10.1016/j.htct.2024.05.009

**Published:** 2024-08-21

**Authors:** Nour Ben Abdeljelil, Rihab Ouerghi, Insaf Ben Yaiche, Amine Ben Moussa, Yosra Chebbi, Tarek Ben Othman

**Affiliations:** a Centre National de Greffe de Moelle Osseuse de Tunis. Service d'Hématologie et de Greffe, Tunis, Tunisia; b Université de Tunis El Manar, Faculté de Médecine de Tunis, Tunis, Tunisia; c Centre National de Greffe de Moelle Osseuse de Tunis. Service des Laboratoires, Tunis, Tunisia

**Keywords:** Allogeneic hematopoietic stem cell transplantation, Bacteremia, Antimicrobial resistance, Risk factors, Mortality

## Abstract

**Objective:**

Bacteremia is a serious complication in patients undergoing allogeneic hematopoietic stem cell transplantation. The aim of this study was to determine the frequency, epidemiological profile, and risk factors of bacteremia early after allogeneic hematopoietic stem cell transplantation.

**Methods:**

An observational descriptive retrospective study was conducted in patients who received transplants between January 2016 and December 2021. Early bacteremia was defined as blood stream infection occurring between Day 0 and Day 100 after transplantation.

**Results:**

Forty episodes of early bacteremia occurred in 36/245 transplanted patients. Fifteen episodes (37.5%) were due to gram-positive bacteria and 25 (62.5%) to gram-negative bacteria. The most frequent species isolated were coagulase negative staphylococci (CoNS) in gram-positive bacteremia (n = 8/15), and *Klebsiella* species (8/25) and *Pseudomonas* species (8/25) in gram-negative bacteremia. Twenty-nine episodes of bacteremia (72.5%) occurred during the first 30 days after transplantation with a median time of nine days (range: 0-90 days). Coagulase negative staphylococci were methicillin-resistant in 75% of cases, the only *Staphylococcus aureus* isolated was methicillin-resistant. All gram-positive bacilli were penicillin-resistant. Gram-negative bacilli were multidrug resistant in 61.5% of cases. In multivariate analysis, bone marrow as source of graft (p-value = 0.02) and cytomegalovirus reactivation (p-value = 0.02) were significantly associated with an increased risk of bacteremia. Mortality attributable to bacteremia was 2.8%. The one-year overall survival was not significantly different between those with and without bacteremia.

**Conclusions:**

Bacteremia was more frequent within the first 30 days after transplantation indicating the crucial role of neutropenia. An increase in multidrug resistant gram-negative bacteremia was noted.

## Introduction

Patients undergoing allogeneic hematopoietic stem cell transplantation (allo-HSCT) are at high risk of infectious complications due to prolonged neutropenia, mucosal disruption from the conditioning regimen, graft-versus-host disease (GvHD) and corticosteroid therapy.^1^ Bacteremia documented in blood cultures, which occurs in roughly 20% of patients undergoing allo-HSCT,^2^ is associated with increased risk of mortality.^1^ Early detection and prompt use of antibiotics are crucial to reduce bacteremia-related mortality. Over the past decade, gram-positive (GP) cocci were the most common organism isolated in blood cultures in HSCT recipients.^3^ A shift has been reported in recent studies with the emergence of gram-negative (GN) bacilli and the spread of multidrug-resistant (MDR) GN bacteria.^4^^,^^5^ The aim of this study was to determine the frequency, epidemiology, and risk factors of bacteremia occurring within the first 100 days after allo-HSCT with matched sibling donors (MSD) without the use of antibiotic prophylaxis and the impact of bacteremia on survival.

## Methods

### Patient population

A Single center descriptive retrospective cohort study was conducted in patients who underwent allo-HSCT from January 2016 to December 2021. Only human leukocyte antigen (HLA) MSD allo-HSCT are performed in the center of this study. Patients with at least one positive blood culture for significant bacteria within 100 days after allo-HSCT were included in the bacteremia cohort and all the others were included in the non-bacteremia cohort. Clinical and biological data were collected from the patient's medical records.

### Supportive care

All patients had double central venous catheter (CVC) insertion before starting the conditioning regimen.

Screening for MDR bacteria was performed with weekly deep rectal swabs before the start of conditioning and until hospital discharge to detect extended-spectrum beta-lactamase (ESBL), carbapenemase producers and vancomycin-resistant enterococci (VRE). First-line antibiotic treatment consisted of piperacillin-tazobactam and amikacin in the absence of colonization by MDR GN bacteria. Second-line therapy consisted of antifungal therapy (caspofungin or voriconazole). Glycopeptide was used empirically as a second-line therapy in patients with evidence of CVC or skin infections, mucositis Grade IV, and colonization by *staphylococcus* or *streptococcus* resistant to piperacillin-tazobactam.

Nasal and sputum swabs were performed weekly for *Aspergillus sp.* as screening tests for invasive aspergillosis. Patients were monitored once a week from engraftment to Day +100 for cytomegalovirus (CMV) using the pp 65 antigenemia test or by quantitative polymerase chain reaction (PCR). All patients received prophylactic fluconazole (400 mg/day in adults and 6 mg/kg/day i.v. in children) from Day -7 to Day +75 and acyclovir as prophylaxis against the herpes simplex virus (HSV) from Day +1 to Day +180. No antibiotic prophylaxis was used.

All patients were transfused with phenotyped, leukodepleted, and irradiated red blood cells, and with leukodepleted and irradiated single donor platelets or random donor platelets. Patients received granulocyte colony-stimulating factor (G-CSF -5 mg/kg/day) from Day +7 until the neutrophil count reached 1 × 10^9^/L on three consecutive days. Engraftment was defined as the first of three consecutive days with an absolute neutrophil count higher than 0.5 × 10^9^/L.^6^ All patients were treated in laminar airflow isolation rooms with high-efficiency particulate air filtration and received gut decontamination with oral colistin, gentamicin and fungizone.

### Blood-culture sampling

A blood culture was performed in the presence of systemic symptoms of infection (fever, hypotension or malaise). Surveillance blood cultures were carried out in asymptomatic patients during treatment with steroids to detect occult bacteremia. Blood samples for aerobic and anaerobic growth conditions, from central lines and concomitant blood samples from a peripheral vein for blood cultures were routinely performed. C-reactive protein (CRP) was monitored every other day.

### Bacteremia

Bacteremia was defined as the isolation of bacteria from at least one blood culture. For coryneforms or coagulase negative staphylococci (CoNS), two consecutive positive blood cultures were required. Early bacteremia was considered as occurring between Day 0 and Day +100 after allo-HSCT. Bacteremia was considered catheter-related when the causative microorganism isolated in the blood was also recovered from a catheter hub or tip, or a positive culture of a blood sample from CVC was found at least two hours earlier than a positive result of a peripheral blood culture.^7^ A repeat bacteremic episode was considered when the same microorganism was isolated after an interval of more than seven days from the earlier episode with adequate treatment or when a different microorganism was isolated after an interval of more than three days from the earlier episode.^8^ Polymicrobial bacteremia was defined as the isolation of more than one microorganism from the same blood sample or from two consecutive blood samples within 24 hours.^9^

### Identification and antimicrobial susceptibility testing

Blood samples were analyzed according to the “Référentiel en Microbiologie Médicale”.^10^ Bacterial identification was based on morphologic, cultural and biochemical characteristics (Api systems, BioMérieux®). Antimicrobial susceptibility testing was performed by the diffusion method on agar medium according to the Comité de l'Antibiogramme de la Société Française de Microbiologie (CAS-FM) standards.^11^ The minimal inhibitory concentrations (MIC) of colistin for ESBL producing Enterobacteriales (ESBL-E), MDR *P. aeruginosa* and MDR *A. baumannii* were performed using the microdilution method (Biocentric®). The MIC of glycopeptides for methicillin-resistant *S. aureus* (MRSA) and VRE were determined by the microdilution method (Biocentric®) and E-test (BioMérieux®), respectively. ESBL identification was determined by the double disk synergy test. MDR bacteremia was defined as the isolation in the blood of MDR bacteria (ESBL-E, *P. aeruginosa* or *A. baumannii* resistant to at least three families of antibiotics - β-lactam, aminoglycoside, fluoroquinolone, colistin, - MRSA and VRE).

### Antibiotic treatment

Bacteremia was initially treated by empiric broad-spectrum intravenous antibiotics. Antibiotics were modified according to the susceptibility of the isolated bacteria and response to treatment. Initial empirical treatment was defined as appropriate if an antibiotic prescribed within 24 hours matched the *in vitro* susceptibility of a pathogen deemed to be the likely cause of infection.^12^ Antibiotic treatment was continued for at least 14 days, although it was continued until the resolution of systemic symptoms in patients with signs of complicated bacteremia, such as the presence of secondary deep infections, persistent bacteremia, and implantable devices.

### Attributable mortality

Attributable mortality was defined as death with persistent signs or symptoms of sepsis, or persistent positive blood cultures, or persistence of a focus of infection with no other reason for death.^13^

### Statistical analysis

Proportions were compared using a Fisher exact test or chi-square test, and continuous variables were compared using a Student t-test or Mann Whitney U test, as appropriate. Acute GvHD was censored at day 100 after allo-HSCT. Overall survival (OS) was defined as the time between allo-HSCT and death from any cause or the last follow-up. Survivor curves were estimated using the Kaplan-Meier method and were compared using the log-rank test. All factors with p-values < 0.2 by univariate analysis were included in multivariable analysis using a Cox regression proportional hazard model with a p-value of less than 0.05 being considered statistically significant.

## Results

### Patient characteristics

Between January 2016 and December 2021, 245 patients received allo-HSCT from MSD. Patient characteristics are detailed in Table 1.

**Table 1:** Patient characteristics of bacteremia and no bacteremia cohorts.

Characteristic	Patients with bacteremia n = 36 (%)	Patients without bacteremia n = 209 (%)	Total n = 245 (%)	p-value
Age				0.9
Children < 18 years	10 (27.8)	59 (28.2)	69 (28.2)	
Adults	26 (72.2)	150 (71.8)	176 (71.8)	
Median age in years (range)	22 (5 – 49)	27 (5 – 56)	26 (5 – 56)	0.9
Sex-ratio (male / female)	2	1.48	1.55	0.42
Diagnosis				0.5
Acute myeloid leukemia	13 (36.1)	69 (33)	82 (33.5)	
Acute lymphoblastic leukemia	11 (30.6)	64 (30.6)	75 (30.6)	
Aplastic anemia	9 (25)	47 (22.5)	56 (22.9)	
Others*	3 (8.3)	29 (13.9)	32 (13)	
Disease status at allo-HSCT				1
CR1	18 (50)	107 (51.2)	125 (51)	
>CR1/failure	7 (19.4)	45 (21.5)	52 (21.2)	
Not applicable *	11 (30.6)	57 (27.3)	68 (27.8)	
HCT-CI score				0.4
0-1	30 (83.3)	186 (89)	216 (88)	
≥2	6 (16.7)	23 (11)	29 (12)	
EBMT score				0.8
0-1	19 (52.8)	114 (54.5)	133 (54.3)	
≥2	17 (47.2)	95 (45.5)	112 (45.7)	
Interval from diagnosis to allo-HSCT, months (range)	7 (1–51)	7 (1–147)	7 (1–147)	0.3
Conditioning intensity				0.7
Non-myeloablative	9 (25)	47 (22.5)	56 (23)	
Myeloablative	27 (75)	162 (77.5)	189 (77)	
Conditioning regimen (%)				0.9
Non TBI-based	30 (83.3)	173 (82.8)	203 (83)	
TBI-based	6 (16.7)	36 (17.2)	42 (17)	
Graft source				0.02
Bone marrow	27 (75)	112 (53.6)	139 (56.7)	
Peripheral blood stem cells	9 (25)	97 (46.4)	106 (43.3)	
Median transplanted cell count				
CMN × 10^8^/kg (range)	1.35 (0.6 – 4.9)	1.8 (0.7 – 4.6)	1.8 (0.6 –4.9)	0.2
CD34^+^ × 10^6^/kg (range)	2.16 (2.1 – 5.4)	4.48 (1.17 – 6.8)	4.40 (2.1 –2.8)	0.5
Median day of PNN engraftment - days (range)	15 (9 –38)	13 (5 – 38)	13 (5 – 38)	0.04
GvHD prophylaxis				0.3
CsA + MTX ± ATG	31 (86)	192 (92)	223 (91)	
CsA only	5 (14)	17 (8)	22 (9)	
Acute GvHD ≥ grade II (n = 243)				0.2
No	21 (58.3)	140 (67.6)	161 (66.3)	
Yes	15 (41.7)	67 (32.4)	82 (33.7)	
CMV infection (n = 243)				0.01
No	19 (52.8)	153 (74)	172 (70.8)	
Yes	17 (47.2)	54 (26)	71 (29.2)	
Corticosteroid therapy				0.04
No	18 (50)	140 (67)	158 (64.5)	
Yes	18 (50)	69 (33)	87 (35.5)	
Length of hospital stay - days (range)	41 (17 – 123)	35 (21 – 134)	36 (17 – 134)	<10^-4^
Median follow-up, (range)	24 m (5d - 81 m)	24 m (4d - 83 m)	24 m (5d - 83 m)	0.7

Others*: Myelodysplastic syndrome; chronic myeloid leukemia; non-Hodgkin's lymphoma; Hodgkin's lymphoma; Primary myelofibrosis; chronic myelomonocytic leukemia; and paroxysmal nocturnal hemoglobinuria; Not Applicable*: Aplastic anemia; Paroxysmal nocturnal hemoglobinuria, MDS without excess blasts and untreated Primary myelofibrosis.

CR: complete remission; TBI: Total body irradiation; CsA: Cyclosporine; MTX: methotrexate; ATG: antithymocyte globulin; CMV: Cytomegalovirus; GvHD: Graft-versus-host disease; CMN: mononuclear cells; allo-HSCT: allogeneic hematopoietic stem cell transplantation; HCT-SCI: Hematopoietic Cell Transplantation-specific Comorbidity Index; EBMT: European Society for Blood and Marrow Transplantation.

### Frequency of early bacteremia

A total of 40 episodes of early bacteremia occurred in 36 of 245 transplanted patients. Overall, 14.6% of transplanted patient were infected with 1.11 episodes per infected patient. Two patients presented two episodes of bacteremia and one patient presented three episodes. One episode of bacteremia was poly-microbial (two different GN bacteria). Fifteen episodes (37.5%) were due to GP and 25 episodes (62.5%) were due to GN bacteria. Forty-one microorganisms were isolated in the 40 episodes of bacteremia. The GP/GN ratio was 0.16. Twenty-nine episodes of bacteremia (72.5%) occurred during the first 30 days after allo-HSCT with the median time from allo-HSCT to bacteremia being nine days (range: 0-90 days). Episodes of bacteremia were catheter-related in 20% of the cases.

### Bacterial isolates

Coagulase negative staphylococci, *Klebsiella* and *Pseudomonas* species were the most common isolated microorganisms in blood cultures. The distribution of the microorganisms isolated in the blood cultures is detailed in Table 2.

**Table2:** Microorganisms isolated in blood cultures.

Microorganism (n = 41)		n (%)
Gram-positive bacteria		**15 (36.6)**

Coagulase-negative staphylococci (CoNS) *Staphylococcus haemolyticus (n = 1)* *Staphylococcus hominis (n = 2)* *Staphylococcus epidermidis (n = 5)*		**8 (19.5)**
*Staphylococcus aureus* Enterococci *Enterococcus faecium (n = 1)* *Streptococcus sp.*		**1** **1** **1**
Gram-positive bacilli *Corynebacterium spp. (n = 2)* *Corynebacterium jeikeium (n = 1)* *Brevibacterium sp. (n = 1)*		**4**

Gram-negative bacteria		**26 (63.4)**

Enterobacteriaceae *Escherichia coli (n = 2)* *Citrobacter freundii (n = 1)* *Klebsiella pneumonia (n = 6)* *Klebsiella oxycota (n = 1)* *Klebsiella aerogenes (n = 1)* *Raoultella terrigenia (n = 2)* *Enterobacter cloacae (n = 1)* *Serratia marcescens (n = 1)*		**15 (36.6)**
Non-fermenting gram-negative bacilli		**11 (26.8)**
*Aeromonas hydrophilia (n = 2)* *Pseudomonas aeruginosa (n = 4)* *Pseudomonas stutzeri (n = 2)* *Pseudomonas putida (n = 1)* *Pseudomonas sp. (n = 1)* *Acinetobacter baumannii (n = 1)*		

### Antimicrobial susceptibility

Coagulase negative staphylococci were methicillin-resistant in 75% of cases (6/8) and all GP bacilli were penicillin-resistant (4/4). The only *enterococcus faecium* isolated was resistant to vancomycin. GN bacilli were MDR in 61.5% of cases (16/26). Of the 15 Enterobacteriaceae isolated, nine were identified as ESBL producers and one case of *Klebsiella pneumonia* was identified as carbapenemase-producing Enterobacteriaceae. Of the 11 non-fermenting GN bacilli, six were identified as MDR (*Pseudomonas* sp. and *Aeromonas* (5/10) and *Acinetobacter baumannii* (1/1)).

The clinical and laboratory characteristics of patients at the time of bacteremic episodes are summarized in Table 3 (all bacteremic episodes were counted in patients who had more than one).

**Table 3:** Clinical and laboratory characteristics of patients at the time of bacteremic episode.

Characteristic	n = 40
Fever - n (%)	39 (97.5)
Concurrent infectious focus - n (%)	18 (45)
Sepsis - n (%)	9 (22.5)
Septic shock - n (%)	3 (7.5)
Neutropenia - n (%)	31 (77.5)
Neutropenia duration - n (range)	13 days (5-63d)
Absolute neutrophil count/µL - n (range)	60 (0 – 10 710)
Central venous catheter - n (%)	39 (97.5)
Median duration of central venous catheter	36 days (8 –80d)
Mucositis Grade III-IV - n (%)	24 (60)
Duration of Grade III-IV mucositis - n (range)	9 days (0 –25d)
Parenteral nutrition - n (%)	22 (55)
Median duration of parenteral nutrition - n (range)	10 days (0 – 30d)
Median CRP value in mg/L - n (range)	65 (2 – 259)
Concurrent CMV infection(s) - n (%)	17 (42.5)
Acute GvHD ≥ grade II - n (%)	17 (42.5)
Corticosteroid therapy - n (%)	20 (50)

CMV: Cytomegalovirus; CRP: C-reactive protein; GvHD: Graft versus host disease

Concurrent infectious focuses were present in 18 patients (45%), including pneumonia (n = 6), enterocolitis (n = 4) and catheter-related bloodstream infections (n = 8) due to CoNS (n = 4), *Pseudomonas aeruginosa* (n = 1), *Raoultella terrigenia* (n = 1), *Serratia marcescens* (n = 1), and *Corynebacterium jeikeium* (n = 1). Prior rectal colonizations by MDR bacteria were detected in 14 patients (35%) within the month prior to bacteremia. *Klebsiella pneumoniae* was the most common microorganism isolated (seven patients) followed by *Escherichia coli* (four patients) and *Enterococcus faecium* (two patients). Of the 14 patients previously colonized by MDR bacteria, the bacteria detected in blood cultures were the same as the colonizing bacteria in six patients. Three patients had septic shock due to *E. coli, Raoultella terrigenia*, and *P. aeruginosa*. Corticosteroid therapy was prescribed in 50% of patients for GvHD.

At the time of the bacteremic episode, 30 patients (75%) had an elevated CRP (>5mg/L). Procalcitonin and lactate were not routinely assessed during the study period.

### Treatment

Median time between the onset of antibiotic therapy and the first positive blood culture was six days. Sixteen episodes (40%) occurred during antibiotic therapy prescribed for fever of unknown origin. The episode of bacteremia was a new infectious episode in these patients and the cultures were collected at the same time that the antibiotics were started for each infectious episode. Antibiotics were appropriate in 57.5% of the cases of bacteremia (23/40). Initial antibiotic therapy was modified in 50% of cases (n = 20): inappropriate (n = 16) and used for synergistic effect (n = 4). Two patients had methicillin-resistant CoNS bacteremia with a favorable outcome with piperacillin-tazobactam. Teicoplanin was the most frequently prescribed antibiotic (n = 21) followed by imipenem (n = 19) and piperacillin-tazobactam (n = 13). The median duration of treatment for bacteremia was 14 days (range: 9-26 days). One patient died after nine days following bacteremia due to septic shock. The CVC was removed in eight patients (20%), four of whom had catheter-related bacteremia. The CVC was removed within an average of five days of bacteremia (range: 1-8 days) and within three days (range: 1-4 days) in patients with appropriate antibiotic therapy. Catheter removal and antibiotic therapy contributed to infection control in seven bacteremic episodes (17.5%).

### Risk factors of early bacteremia after allo-HSCT

In multivariate analysis, the use of bone marrow as graft source and CMV reactivation were significantly associated with early bacteremia after allo-HSCT in the present study (Table 4).

**Table 4:** Risk factors of early bacteremia after allo-HSCT.

Variable	Patients with bacteremia n = 36 (%)	Patients without bacteremia n = 209 (%)	Univariate analysis p-value	Multivariate analysis OR (CI 95%)	p-value
Age < 40 years - n (%)	31 (15.6)	168 (84.4)	0.4	-	-
Age ≥ 40 years - n (%)	5 (10.9)	41 (89.1)			
Gender of patient - n (%)					
Male	24 (16.1)	125 (83.9)	0.4	-	-
Female	12 (12.5)	84 (87.5)			
Diagnosis - n (%)			0.5		
Non-malignant disease	11 (16.6)	54 (83.4)		- -	
Malignant disease	25 (14)	155 (86)			
Disease status at allo-HSCT - n (%)					
CR1	18 (14.4)	107 (85.6)	1	-	-
>CR1/failure	7 (13.4)	45 (86.6)			
Not applicable*	11 (16.2)	57 (83.8)			
EMBT score - n (%)					
0-1	19 (14.4)	114 (85.6)	0.8	**-**	-
> = 2	17 (23.3)	56 (76.7)			
Conditioning regimen - n (%)					
Non TBI-based	30 (14.8)	173 (85.2)	0.9	**-**	-
TBI-based	6 (14.3)	36 (85.7)			
Graft source - n (%)					
Peripheral blood stem cells	9 (8.5)	97 (91.5)	**0.02**	**2.61 (1.15–5.9)**	0.02
Bone marrow	27 (19.4)	112 (80.6)			
ESBL/MDR/VRE colonization - n (%)					
No	24 (15.8)	128 (84.2)	0.5	**-**	-
Yes	12 (12.9)	81 (87.1)			
Neutropenia duration >13 days- n (%)					
No	21(13)	139 (87)	0.3	**-**	-
Yes	15 (17.7)	70(82.3)			
Grade III-IV mucositis duration > 9 days - n (%)					
No	22 (17.5)	104(82.5)	0.22	-	-
Yes	14 (11.8)	105(88.2)			
Parenteral nutrition duration >10 days - n (%)					
No	17 (15.6)	92(84.4)	0.7	-	-
Yes	19 (14)	117(86)			
Catheter duration >36 days - n (%)					
No	18 (11.3)	142 (88.7)	**0.033**	NS	NS
Yes	18 (21.4)	66 (78.6)			
Acute GvHD ≥ Grade II - n (%)					
No	21 (13)	140 (87)	0.27	-	-
Yes	15 (18.3)	67 (81.7)			
CMV infection(s) - n (%)					
No	19 (11)	153 (89)	**0.01**	**2.3 (1.09-4.85)**	0.02
Yes	17 (24)	54 (76)			
Corticosteroid therapy - n (%) (within the first 100 days)					
No	18 (11.4)	140 (88.6)	**0.04**	NS	NS
Yes	18 (20.7)	69 (79.3)			

OR: Odds ratio; 95% CI: 95% confidence interval; allo-HSCT: Allogeneic hematopoietic stem cell transplantation; ESBL: extended-spectrum β-lactamase; MDR: Multidrug-resistant Bacteria; VRE: vancomycin-resistant Enterococcus; TBI: Total body irradiation; CR: complete remission; Not applicable*: Aplastic anemia, Paroxysmal nocturnal hemoglobinuria, Myelodysplastic syndrome without excess blasts and untreated Primary myelofibrosis; CMV: Cytomegalovirus; EMBT: European Society for Blood and Marrow Transplantation; GvHD: Graft versus host disease

### Overall survival

After a median follow-up of 24 months (range: 5 days-83 months), the overall survival was 80.4% in patients with early bacteremia and 78.7% in patients without early bacteremia (p-value = 0.8).

### Mortality

Of the 245 transplanted patients, 53 (21.6%) patients died (26 from relapse and 27 from non-relapse mortality. The non-relapse mortality was 11.1% (4/36) in patients with bacteremia and 11% (23/209) in patients without bacteremia (p-value = 0.72). Attributable mortality was 2.8% (1/36); caused by an MDR-*Raoultella terrigena* bacteremia despite appropriate antibiotic therapy and CVC removal.

## Discussion

This study, conducted in a large number of patients who underwent allo-HSCT from MSD between 2016 and 2021, provides data on the frequency, antimicrobial resistance and management of early bacteremia following allo-HSCT without the use of prophylactic antibiotics in a single center.

Bacteremia is a common infectious complication of patients undergoing allo-HSCT and continues to be a major cause of mortality. It usually occurs in the pre-engraftment period. Neutropenia, mucosal barrier injury caused by conditioning regimens, GvHD and corticosteroid therapy increases host susceptibility to bacteremia. In the present study, 14.6% of patients developed early bacteremia after allo-HSCT. The incidence of early bacteremia after allo- HSCT ranged from 7-50% in previous studies.^14^, ^15^, ^16^ A recent meta-analysis conducted by Mikuslka et al. found that prophylaxis with fluoroquinolones was associated with a significantly lower incidence of bacteremia but had no impact on overall mortality.^17^ In the current center, routine antibiotic prophylaxis is not carried out. Despite the lack of demonstrated benefit in adding aminoglycosides to beta-lactam therapy compared to beta-lactam monotherapy as reported in a systematic review and meta-analysis in 2003,^18^ we adopt empirical beta-lactam-aminoglycoside combination therapy as frontline treatment for febrile neutropenia due to the emergence of MDR bacterial strains accentuated by our department's ecology. Additionally, in severe cases, particularly septic shock, easy access to intensive care services is not readily available. We adapt first line empirical antibiotic therapy to the bacteria isolated in the bloodstream during the first courses of chemotherapy and to bacteria isolated in periodic rectal swabs during the early phase of allo-HSCT. In this study, only early bacteremia was analyzed, which might explain the relatively low rate of bacteremia observed. Increasing the use of peripheral blood stem cells as the graft source during the study period led to a reduction in the duration of neutropenia, which also might have decreased the incidence of bacteremia. Most of the episodes of bacteremia occurred within the first 30 days after allo-HSCT, which may be related to neutropenia. In the remaining cases (after 30 days), episodes of bacteremia occurred in patients who had developed GvHD, either under corticosteroid therapy, or following CMV reactivation.

Gram-negative bacteria were the most frequently isolated microorganisms in this study (63.4%) both within the first 30 days and after 30 days of allo-HSCT, mostly due to the *Pseudomonas* and *Klebsiella* species. *E. coli* was the most common microorganism in most studies.^16^^,^^19^ A number of studies have previously reported that GPs are the most predominant microorganisms.^20^, ^21^, ^22^ The use of fluoroquinolone prophylaxis in some centers, differences between conditioning regimen, and increased use of CVCs probably contributed to the increased rates of GP bacteremia.^20^^,^^23^

Antimicrobial resistance is a major threat to transplanted patients with bacteremia and presents a challenge to infection control in this population. The rise of MDR bacteremia has already led to a substantial increase in the death rate.^24^^,^^25^ Several studies showed that methicillin resistance of CoNS strains isolated from blood cultures are increasing.^20^^,^^26^ The prevalence of MDR bacteremia was more than 90% in a study conducted by Yan et al. ^27^ and 30.1% in a study conducted by Wang et al. ^28^. In recent years, the emergence of CPE bacteremia is remarkably alarming. *Klebsiella pneumoniae* accounted for the majority of CPE isolates.^24^^,^^25^ The threat of CPE is substantial as carbapenems are generally used to treat infections caused by ESBL-E,^29^ and are increasingly being used in the empirical treatment in ESBL-E colonized patients. Consequently, their use has probably contributed to the spread of carbapenem resistance by selective pressure on the gut microbiota.^30^^,^^31^ A high rate of MDR was observed in this cohort. The profile of this patient population, predominantly with acute leukemia and aplastic anemia, who had previously received broad-spectrum antibiotic therapy before allo-HSCT, may have contributed to the emergence of MDR GN bacteria which led to increased antibiotic overuse and facilitated the selection of these pathogens and their spread, in addition to transplant-related factors such as corticosteroid therapy and GvHD.

The data on bacterial resistance profiles in the current center showed an emergence of MDR bacteria, which remained stable over the period from 2016-2021. The methicillin resistance rate of *S. epidermidis* responsible for infections was 70% in 2020. In the same year, the rates of ESBL and carbapenemase-producing Enterobacteriaceae were 20% and 9%, respectively. The resistance rates to piperacillin-tazobactam and imipenem for all GN bacteria were 35% and 15%, respectively (data not published).

Elevation of CRP is used as an early marker to predict severe infection.^32^ The CRP level was monitored every other day in the current cohort in order to detect infection in asymptomatic patients and to evaluate the response to antibiotic therapy in patients with severe infections. The assessment of procalcitonin and lactate was not normally practiced during the study period. The CRP level is a limited predictor factor of infection and should not be used as the only parameter to initiate antibiotic therapy as this biomarker can be elevated for reasons other than infection, especially in patients undergoing allo-HSCT, such as due to the conditioning regimen, mucositis, GvHD, and engraftment syndrome. There is no single marker capable to distinguish infection from other complications. Nevertheless, a sudden increase in CRP levels in post-allo-HSCT patients should always be closely assessed, as it may indicate the onset of a potentially life-threatening complication.^33^^,^^34^

Predisposing risk factors for early bacteremia in this cohort were bone marrow as a graft source and CMV reactivation. Patients, with CMV reactivation are at high risk of mortality due to bacterial and fungal infections.^35^ CMV is associated with neutropenia because of a direct viral infection or ganciclovir administration. Additionally, CMV gastrointestinal disease often causes severe inflammatory symptoms, including deep ulcerations which can lead to bacterial and fungal infections. Apart from organ involvement, CMV reactivation may trigger indirect effects such as immunosuppression and increased risk of GvHD, potentially leading to the development of concurrent infectious complications.^36^^,^^37^ Additional risk factors for early bacteremia have been described in the literature, including prolonged neutropenia, mucositis, and the presence of a CVC. The type of underlying disease, severe GvHD, and corticosteroid therapy are also considered major risk factors contributing to early bacteremia after allo-HSCT.^38^ Niyazi et al. reported that fecal colonization with MDR bacteria prior to allo-HSCT and pre-transplant bacteremia are independent risk factors for early bacteremia.^39^

The optimal appropriate treatment duration of bacteremia has been poorly defined. A meta-analysis of bacteremic patients receiving shorter (5-7 days) versus longer (7-21 days) antibiotic therapy found no significant difference in clinical cure, microbiological cure or survival.^40^

The Infectious Diseases Society of America guidelines for the treatment of bloodstream infections suggest seven days of intravenous antibiotics should be given for uncomplicated GN bloodstream infections and catheter-related CoNS (with CVC removal) and 14 days for complicated GN and *Staphylococcus aureus* bloodstream infections.^7^

In the current institution, we adhere to a 14-day treatment duration for bacteremia as we do not systematically remove the CVC, and also given the prevalence of MDR microorganisms in the majority of patients and the restricted access to intensive care services in case of clinical deterioration.

Overall survival of patients with and without bacteremia was comparable in this cohort. Overall survival has been reported as significantly lower in patients with bacteremia compared to those without bacteremia.^16^^,^^41^ Other transplant-related complications, including chronic GvHD and subsequent opportunistic infections may explain this finding.^42^ Ge et al. reported that the overall survival of patients with MDR bacteremia was significantly lower than that of patients without MDR bacteremia.^15^ Attributable mortality to early bacteremia was low in the current cohort. The absence of significant differences regarding overall survival between patients with and without bacteremia and the low attributable mortality of early bacteremia in these patients may be explained by the start of appropriate empiric antibiotic therapy and the empirical addition of glycopeptide antibiotics as second line to prevent *streptococci* infections.

The present research has several limitations. First, it is not a case-control study. This makes it difficult to attribute any causal effect in this setting. In addition, this study was performed in a single-center, which could lead to a biased microbial ecology. The short duration of the study and the relatively small sample size make it difficult to extrapolate outcomes to a larger transplanted patient cohort. Furthermore, identification of portal of entry of bacteria is difficult to determine retrospectively. Rather, we stipulated to identify a concurrent focus of infection. The relationship between changes in patient colonization and repeated exposure to broad-spectrum antibiotics before allo-HSCT was not evaluated in this cohort, which could explain the emergence of MDR bacteremia.

## Conclusions

GN bacteremia remains the predominant causative microorganism of bloodstream infection after allo-HSCT. Reevaluating empiric antibiotic therapy for neutropenic fever, monitoring of antimicrobial resistance and prevention of nosocomial transmission are essential.

## Source of funding

This study did not receive any specific grant from funding agencies.

## Ethical approval

There are no ethical issues.

## Authors contributions

NBA conceived and designed the study, conducted research, provided research materials, collected and organized data and wrote initial and final drafts of article. RO analyzed and interpreted data and provided logistic support. IBY interpreted data for the study and provided logistic support. ABM collected and organized data. YC analyzed and interpreted data. TBO approved final version to be published and agreed to be accountable for all aspects of the work.

All authors have critically reviewed and approved the final draft and are responsible for the content and similarity index of the manuscript.

## Conflicts of interest

The authors have no conflict of interest to declare.
